# Effectiveness and impact of intravenous magnesium sulfate in spinal surgery systematic review and meta-analysis

**DOI:** 10.3389/fphar.2025.1624119

**Published:** 2025-06-18

**Authors:** Zhaoguo Jin, Jianyong Zhao

**Affiliations:** First People’s Hospital of Linping District, Hangzhou, China

**Keywords:** magnesium sulfate, spinal surgery, meta-analysis, postoperative pain, opioid consumption, systematic review, anesthesia

## Abstract

**Background:**

Effective pain management following spinal surgery is crucial for preventing complications related to delayed mobilization. Magnesium sulfate (MgSO_4_) has shown promise as an analgesic agent, influencing neurotransmitter modulation and autonomic nervous system regulation. However, studies evaluating its effectiveness and safety in spinal surgery remain inconsistent, necessitating a comprehensive meta-analysis to assess its role.

**Objective:**

This study aimed to perform a systematic meta-analysis to compare the safety and efficacy of magnesium sulfate against standard therapeutic options in spinal surgery.

**Methods:**

The meta-analysis followed PRISMA guidelines. We performed data extraction and analysis using Review Manager version 5.4. The study population included patients undergoing spinal surgery, with the intervention group receiving intravenous magnesium sulfate at varying dosages or in combination with other agents. The comparison group received either a placebo or alternative treatments. Primary outcomes included pain intensity, opioid consumption, and safety parameters.

**Results:**

Ten randomized controlled trials involving 641 patients were included. Magnesium sulfate administration significantly reduced pain scores at 24 h (MD −0.18, 95% CI: −0.34 to −0.02) and decreased opioid consumption (SMD −0.34, 95% CI: −1.07 to −0.35). Additionally, a significant reduction in muscle relaxant usage was observed (SMD −0.91, 95% CI: −0.66 to −0.10). When compared with dexmedetomidine, magnesium sulfate improved verbal response (MD 1.22, 95% CI: −0.16–2.61) and prolonged extubation time (MD 0.91, 95% CI: −0.98–2.80). No significant differences in hemodynamic parameters (heart rate and blood pressure) were observed between the groups.

**Conclusion:**

Intravenous magnesium sulfate demonstrated significant benefits in reducing postoperative pain and opioid consumption, while also improving verbal response and orientation. These findings suggest that magnesium sulfate may serve as a valuable adjunct in the perioperative management of spinal surgery patients. Further research is required to confirm these results and establish optimal dosing protocols.

## 1 Introduction

Chronic low back pain represents a significant burden on healthcare systems worldwide (Shetty et al., 2022). The management of this condition requires a stepwise, progressive approach that prioritizes conservative treatments, including physiotherapy and lifestyle modifications. However, in severe and refractory cases, invasive interventions such as epidural steroid injections or surgical procedures may become necessary (Ketenci and Zure, 2021). While epidural steroid injections have demonstrated Level I evidence in treating radicular pain associated with disc herniation, concerns persist regarding their safety due to rare but potentially severe neurological complications (Helm et al., 2021).

Within this therapeutic landscape, various interventions for low back pain have been investigated. Ozone therapy, for instance, has shown promise in pain relief, though it remains controversial due to limited long-term evaluation and unclear mechanisms of action (de Andrade et al., 2019). Extensive research has been conducted on adjuvant medications in spine surgery, particularly focusing on ketamine and gabapentin use in adolescents undergoing spinal fusion for idiopathic scoliosis. These trials primarily evaluated the medications’ effectiveness in reducing postoperative pain and minimizing opioid consumption. Notably, these studies have demonstrated significant reductions in both opioid use and pain intensity during the initial 48 postoperative hours, accompanied by favorable safety profiles and low adverse event rates (Bas et al., 2023; Mariscal et al., 2022).

Postoperative pain management remains a critical concern for both clinicians and patients, as delayed mobilization can lead to serious adverse outcomes (Naftalovich et al., 2022). The impact of acute pain has been well-documented in numerous studies. Multiple therapeutic modalities, encompassing both pharmacological and non-pharmacological approaches, have been employed to address postoperative pain, which significantly influences patient prognosis and treatment outcomes (Mentes et al., 2008).

Magnesium, an inorganic ion, has diverse therapeutic applications, including the management of asthma exacerbations, hypokalemia, premature labor, myocardial protection following ischemia, postoperative acute pain control, and hemodynamic stabilization during intubation (Lysakowski et al., 2007). Among magnesium preparations, magnesium sulfate has gained particular attention, with its value being evaluated in numerous anesthesia-related investigations (Buvanendran et al., 2002; Ferasatkish et al., 2008). The anti-nociceptive properties of magnesium (Mg) are attributed to its antagonistic effect on N-Methyl-D-aspartate (NMDA) receptors. While preoperative magnesium administration has shown limited impact on postoperative pain, several clinical trials have demonstrated that magnesium infusion during general anesthesia reduces both anesthetic requirements and postoperative analgesic consumption (Ünlügenç et al., 2002). However, the effects of magnesium sulfate administration during regional anesthesia remain incompletely characterized.

Current evidence regarding magnesium sulfate use in spinal surgery is both conflicting and incomplete. While some studies have reported favorable outcomes, including improved hemostatic parameters, reduced intraoperative bleeding, and enhanced pain scores and patient satisfaction (Fathy et al., 2022; James et al., 1989), others have documented adverse effects such as prolonged emergence time and delayed recovery (Tsaousi et al., 2020). Despite these contradictory findings, no meta-analysis has specifically focused on magnesium sulfate use in spinal surgery. Previous literature reviews have noted “low statistical power” in existing studies, highlighting the need for a comprehensive analysis of available data to determine the effectiveness and safety profile of magnesium sulfate in spinal surgery procedures.

Therefore, this study aimed to conduct a comprehensive meta-analysis to evaluate the efficacy and clinical significance of magnesium sulfate administration in spinal surgery.

## 2 Materials and methods

### 2.1 Eligibility criteria

**FIGURE 1:**
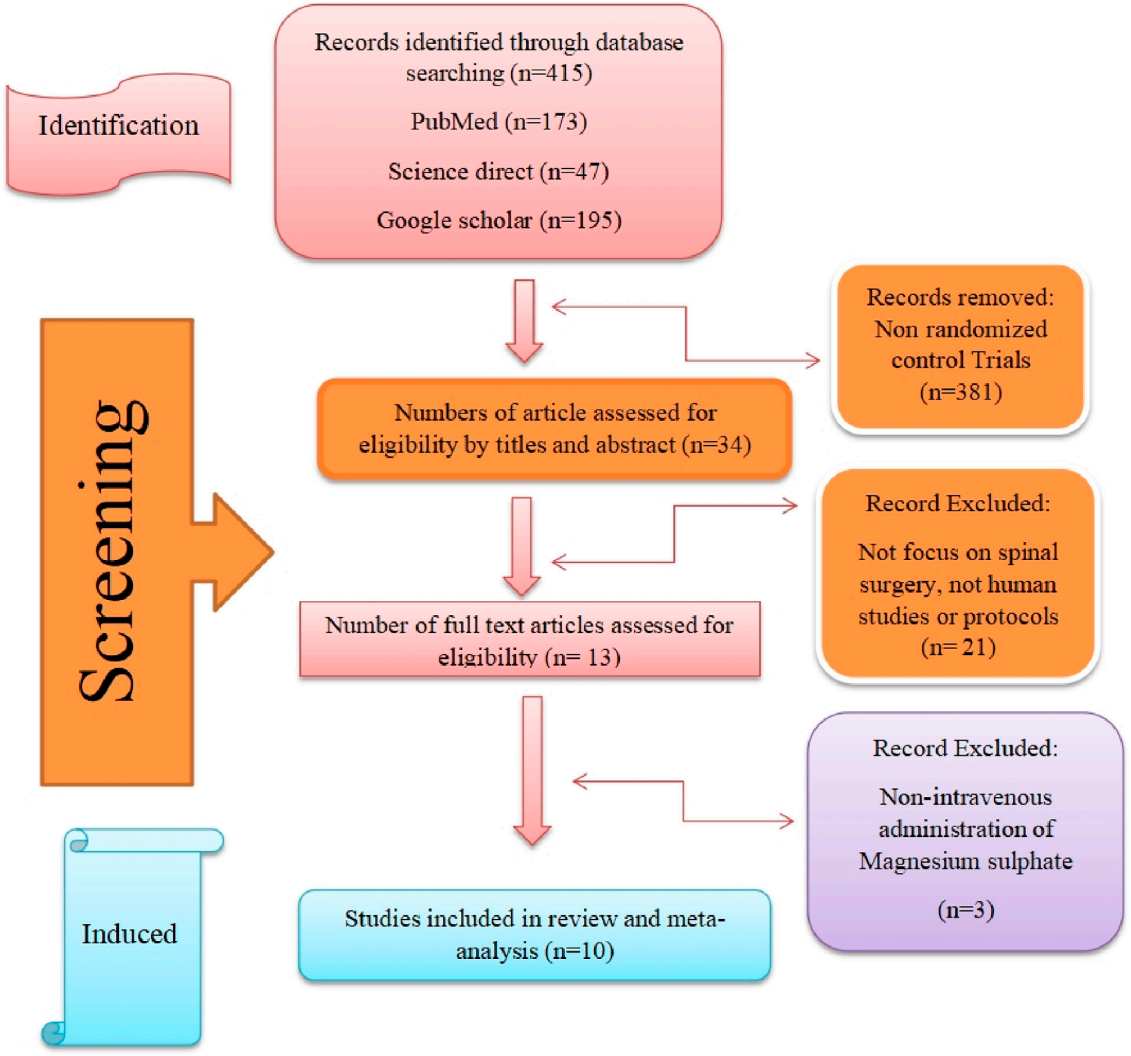
Flow chart of study selection process according to PRISMA.

This meta-analysis was conducted in accordance with the Preferred Reporting Items for Systematic Reviews and Meta-Analyses (PRISMA) guidelines. The study selection process is illustrated in Figure 1 (Tang, 2009). Study inclusion criteria were developed using the PICOS framework. The study population comprised adult patients who underwent spinal surgery. The intervention group received intravenous magnesium sulfate (MgSO_4_) treatment in varying combinations or doses, while the comparison group received either placebo or alternative treatments. Primary outcomes included efficacy metrics (pain management, medication usage, hemodynamic parameters) and safety outcomes determined through complication analysis. Only randomized controlled trials (RCTs) were considered eligible for inclusion.

To minimize bias and prevent data duplication, studies with the following characteristics were excluded:

• Duplicate publications
• Non-randomized studies, due to their inherent limitations in establishing causal relationships and controlling for confounding variables
• Review articles, as they represent secondary analyses rather than primary data
• Studies with incomplete or missing data, to ensure result accuracy and consistency
• Studies that did not share variables with at least two other included studies, to enable meaningful data pooling and robust statistical analysis

### 2.2 Information sources and search strategy

A comprehensive literature search was conducted across multiple databases without language or publication date restrictions. The final search was performed in October 2023, encompassing PubMed, Google Scholar, ScienceDirect, and the Cochrane Library. The search strategy employed the Medical Subject Heading (MeSH) terms “Magnesium” and “spine” (detailed search strategy provided in Supplementary Material 1). Additionally, reference lists of included articles were manually screened to identify additional relevant studies.

Study selection was performed independently by two reviewers with Level V expertise (Tang, 2009; Chandler et al., 2019). In cases of disagreement, a third reviewer was consulted to reach consensus. The detailed study selection process is presented in the PRISMA flow diagram (Figure 1).

### 2.3 Data extraction and data items

Data extraction was conducted independently by two reviewers with Level V expertise, with discrepancies resolved through consultation with a third reviewer (Tang, 2009; Chandler et al., 2019). The following data were extracted from each included study:

• Study characteristics (author, location, study duration).
• Patient demographics (mean age, gender distribution).
• Clinical indicators (etiology, spinal drug dosage).
• Primary outcome measures:
o Pain assessment using Visual Analog Scale (VAS).
o Medication usage (opioids, muscle relaxants, remifentanil).
o Hemodynamic parameters (heart rate [HR], mean arterial pressure [MAP]).
o Recovery indicators (extubation time, verbal command response, orientation time).

### 2.4 Risk of bias assessment

**FIGURE 2:**
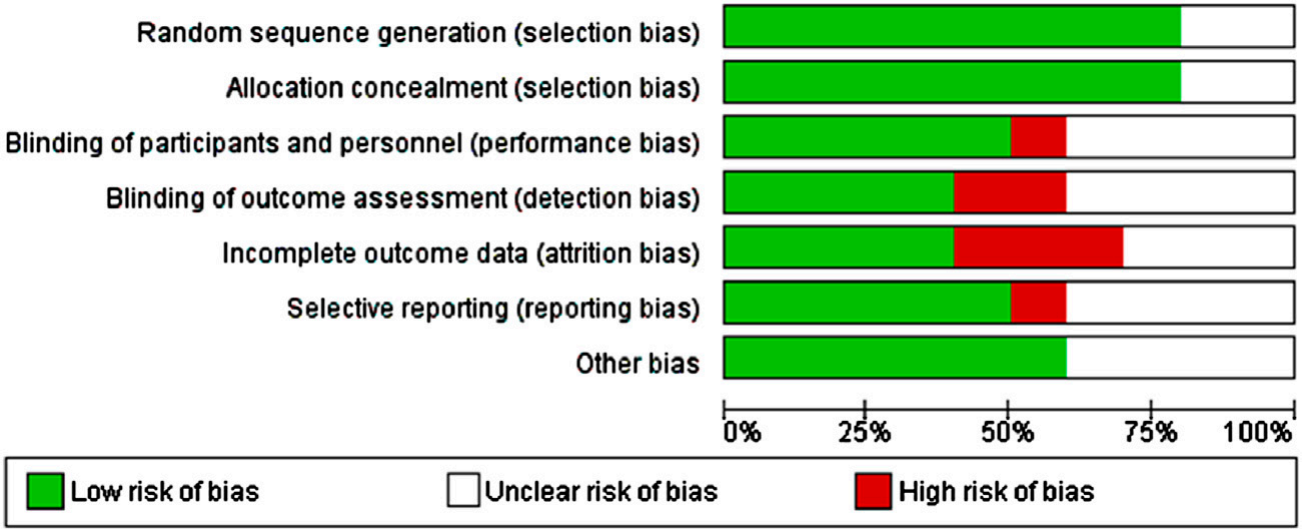
Risk of Bias graph.

The risk of bias in included studies was evaluated using the Cochrane Collaboration’s risk of bias tool and analyzed using Review Manager software (Figure 2). Assessment domains included:

• Random sequence generation.
• Allocation concealment.
• Blinding of participants and personnel.
• Blinding of outcome assessments.
• Incomplete outcome data.
• Selective reporting.

### 2.5 Publication bias

**FIGURE 3:**
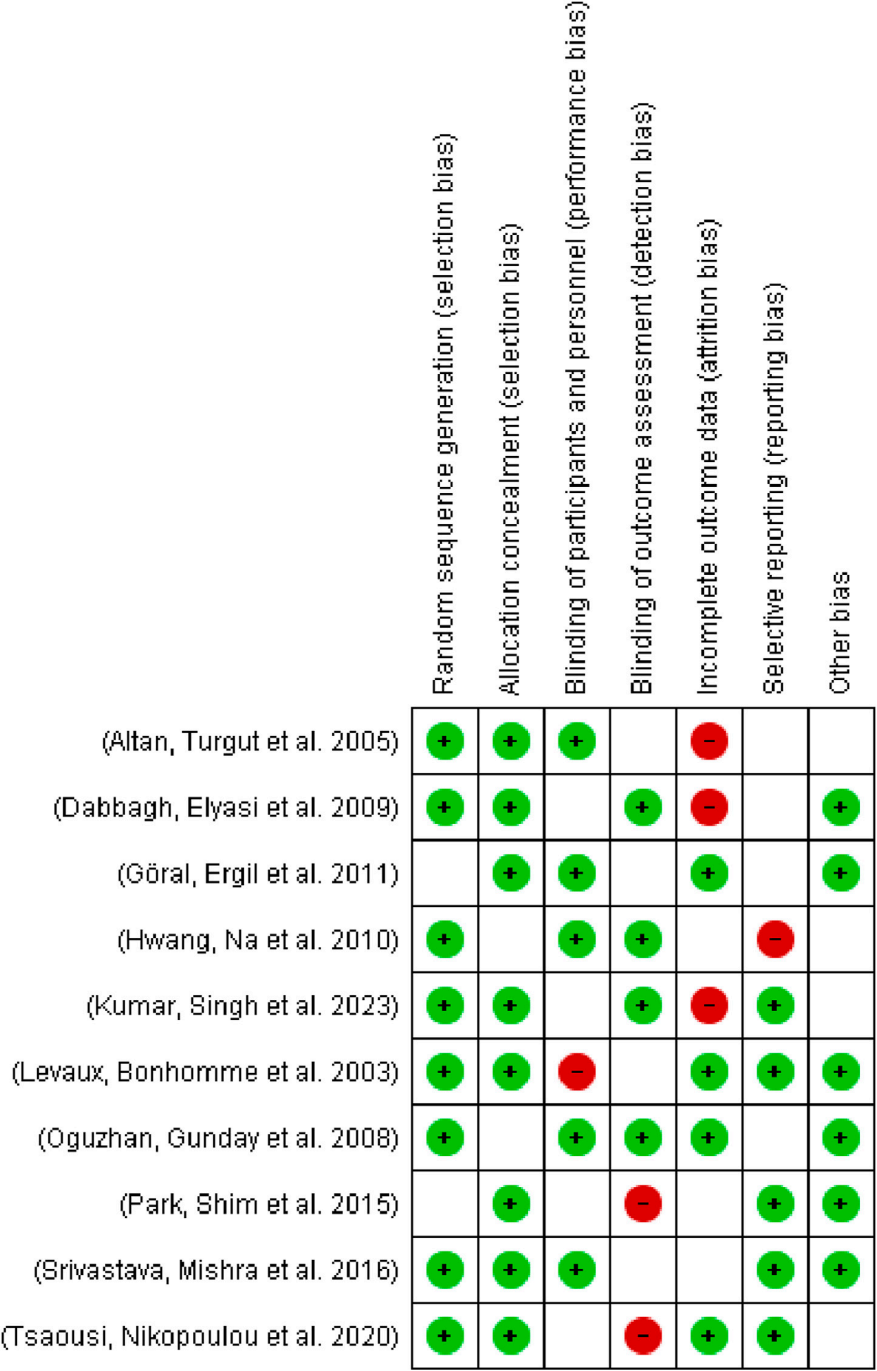
Risk of bias summary.

Review Manager was used to assess publication bias through funnel plot analysis. Funnel plots provide a visual method for examining publication bias by plotting study precision (measured by sample size or standard error) against effect sizes. As shown in Figure 3, the asymmetrical distribution suggests the presence of publication bias, potentially due to the exclusion of smaller studies with non-significant results from the analysis. Additional subgroup analyses were performed to account for multiple time points of outcome measurement, enabling more comprehensive data interpretation.

### 2.6 Statistical analysis

Data analysis was performed using Review Manager 5.4 software. For continuous outcomes, mean differences were calculated, while odds ratios were computed for dichotomous outcomes. All results were presented with 95% confidence intervals. Heterogeneity was assessed using I^2^ and Chi^2^ tests, with I^2^ values > 25%, >50%, and >75% indicating low, moderate, and high heterogeneity, respectively. A fixed-effects model was employed in the absence of significant heterogeneity, while a random-effects model was used when heterogeneity was detected.

Data from figures were extracted using Web Plot Digitizer version 4.5. Missing data were handled according to Cochrane Handbook guidelines (Guyatt et al., 2013). Publication bias was assessed using Review Manager-generated funnel plots, with asymmetrical distribution indicating potential publication bias. Subgroup analyses were conducted to evaluate outcomes at different time points, enabling more detailed data analysis.

## 3 Results

### 3.1 Study selection

The systematic literature search initially identified 415 potentially relevant studies. After applying the clinical trial filter, 381 studies were excluded, leaving 34 studies for further evaluation. Following title and abstract screening, 21 studies were excluded based on predefined criteria: they were either study protocols, non-human studies, pharmacokinetic studies, or studies not focused on spinal surgery.

Of the remaining 13 studies that underwent full-text review, three were subsequently excluded due to:

• Non-shared outcome variables
• Duplicate publications
• Non-intravenous magnesium sulfate administration routes
• Significant heterogeneity in inclusion criteria

Manual screening of reference lists from included studies did not yield any additional eligible articles. Therefore, ten randomized controlled trials were ultimately included in the meta-analysis, as illustrated in the PRISMA flow diagram (Figure 1).

### 3.2 Study characteristics

The primary characteristics of the included studies are summarized in Table 1. The meta-analysis comprised ten studies with a total of 641 participants: 295 in the magnesium sulfate (MS) group, 163 in the placebo group, 103 in the dexamethasone group, and 80 in the dexmedetomidine group. The mean age of participants ranged from 32.2 to 55.9 years, with 301 participants (46.9%) being female. Table 1 presents the detailed MS dosage regimens and surgical indications for each study. The risk of bias assessment for the included studies is presented in Figure 3.

**TABLE 1:** The main characteristics of included studies.

Study	Region	Dose MgSo4	Surgery site	No. of patients treatment/Placebo	No. of females (MS/PL)	Age	Period
Altan et al. (2005)	Turkey	30 mg/kg 1 over a 15-min period before induction of anesthesia and 10 mg/kg 1 h	Spine surgery	60 (30/30)	7/7	42.3/44.9	NR
Göral et al. (2011)	Turkey	50 mg/kg in 100 mL saline by slow infusion over 10 min, followed by a continuous infusion of 20 mg/kg/h	Single-level microscopic lumbar discectomy	40 (20/20)	10/11	48/49	NR
Kumar et al. (2023)	India	15 mL of 0.75% ropivacaine +500 mg equivalent to 1 mL + 4.0 mL normal saline. The total volume of the solution infiltrated was 20 mL in both groups	Single-level lumbar laminectomy	60 (30/30)	8/5	35.2/38.2	NR
Levaux et al. (2003)	Belgium	50 mg/kg in 250 mL of normal saline over 30 min immediately before induction of anesthesia	Lumbar arthrodesis	24 (12/12)	8/5	55/46	NR
Oguzhan et al. (2008)	Turkey	30 mg/kg (over 10 min) starting immediately after induction of anesthesia and completed before intubation; the infusion was then continued at 10 mg/kg/h throughout surgery	Lumbar disc surgery	50 (25/25)	12/11	44/42	2005 to 2006
Park et al. (2015)	Korea	30 mg/kg in a total of 100 mL normal saline was given for 10 min before the induction of anesthesia, followed by continuous infusion of at 10 mg/kg/h until the end of operation	Lumbar spine surgery	146 (73/73)	32/31	51/51	2013 to 2014
Srivastava et al. (2016)	India	Loading dose 50 mg/kg before induction over a period of 15 min and maintenance 15 mg/kg/h throughout the surgery	Elective spine surgery	90 (45/45)	19/20	48.3/46.6	NR
Tsaousi et al. (2020)	Greece	20 mg/kg diluted in isotonic saline to a volume of 100 mL was infused as an intravenous (i.v.) bolus dose over 15 min before anesthesia induction and thereafter 20 mg/kg/h was continuously infused until surgery completion	Lumbar laminectomy	71 (35/36)	22/21	55.9/49	2020
Hwang et al. (2010)	Korea	magnesium sulphate 50 mg/kg for 15 min and then 15 mg/kg per hour by continuous i.v	spinal surgery	40 (20/20)	11/7	47/49.9	NR
Dabbagh et al. (2009)	Iran	magnesium sulphate dose of 8 mg/kg/h of body weight	Lower limb orthopedic surgery	60 (30/30)	21/24	33.7/35.1	NR

Notes: NR: no data reported.

A comparative analysis of different treatment approaches and anesthesia techniques in spinal surgery pain management is provided in Table 2. This analysis includes various interventions such as esketamine combined with pregabalin, dexmedetomidine, patient-controlled analgesia (PCA), and acupuncture, highlighting their respective outcomes and clinical significance in spinal surgery patients.

**TABLE 2:** A comparative of different treatments and anesthesia TechniquesSpinal in surgery pain management and outcomes.

Author, year	Patient population	Treatment	Operation type	Conclusion
Zhou, (2024)	Spinal cord surgery patients between the ages of 18 and 65	Patients were randomized to receive esketamine (injection dose 0.5 mg· kg-1, infusion dose 0.12 mg· kg-1 · h-1, 48 h after surgery) combined with oral pregabalin (75–150 mg/day, beginning 2 h before surgery and ending 2 weeks after surgery) or equivalent saline and placebo capsules	Moderate-to-severe acute postsurgical pain	Esketamine combined with pregabalin is effective in alleviating APSP after spinal surgery, but analgesic strategies may increase the risk of mild dissociation symptoms
Hwang, (2015)	40 patients undergoing posterior lumbar interbody fusion (PLIF) under general anesthesia	A total of 40 patients underwent posterior lumbar interbody fusion (PLIF) under general anesthesia. Anesthesia was maintained at 3–12 mg/kg/h with propofol, 0.01–0.2 μg/kg/min in remifentanil group and 0.01–0.02 μg/kg/min in dexmedetomidine group, and the bispectral index was maintained between 40 and 60	Total intravenous anesthesia (TIVA)	Dexmedetomidine showed superior efficacy in pain relief and pain management 48 h after PLIF.
Chen, (2022)	Records of successive patients who underwent surgical treatment for degenerative lumbar disease in our hospital from 2013 to 2014	Patients were grouped according to pain control methods, including routine analgesia, patient-controlled analgesia (PCA), and acupuncture. The routine analgesia group took acetaminophen/NSaids and piperidine orally as needed for immediate pain control. The PCA group received a base dose of morphine and a subsequent user-required dose. In the acupuncture group, acupuncture was performed every other day	The procedure included open laminectomy, discectomy, and posterolateral fusion with transpedicle screw internal fixation	For adjunctive pain control after surgery for degenerative lumbar disease, acupuncture may be as effective as traditional analgesia and PCA
Rahimzadeh, (2018)	60 patients aged 15–65 years who were undergoing posterior spinal fusion	A double-blind randomized clinical trial was conducted in 60 patients aged 15–65 years who were to undergo posterior spinal fusion by random sampling. Intraoperative anesthesia was induced and 1% isoflurane was used in both groups. One group was injected with remifentanil by pumping. The experimental group was given dexmedetomidine	Spinal fusion patients	Dexmedetomidine reduced postoperative pain scores and intraoperative bleeding in patients undergoing spinal surgery. The hemodynamic effect of dexmedetomidine group was significantly improved
Umakoshi, (2021)	A total of 156 non-PD patients treated for spinal degenerative diseases and PD patients after spinal surgery from 2013 to 2017	The Hoehn and Yahr scores of D were 8 cases in stage 1, 2 cases in stage 2, 8 cases in stage 3, 10 cases in stage 4, and 0 cases in stage 5. The median daily equivalent dose of levodopa before surgery was 410 mg. Thirteen patients (46%) received subthalamic nucleus (STN) DBS.	Parkinson’s disease	Postoperative pain and functional improvement in PD patients lasted for 2 years, and the complication rate was higher than that in non-PD patients. PD patients with STN DBS maintained good lumbar lordosis 2 years after spinal surgery. STN DBS significantly maintained spinal alignment 2 years after surgery, with pain and improved function
Lee, (2018)	Patients were recruited from a medical center in central Taiwan. Ninety patients participated in the study	Patients who underwent lumbar surgery (n = 86) were randomly assigned to the intervention group (educational intervention; n = 43) or control group (n = 43); Four patients voluntarily withdrew after surgery (1 case in the intervention group; Control group (3 cases)	spinal surgery	Preoperative educational interventions are effective in informing patients undergoing spinal surgery of reduced postoperative pain, anxiety, and fear

**FIGURE 4:**
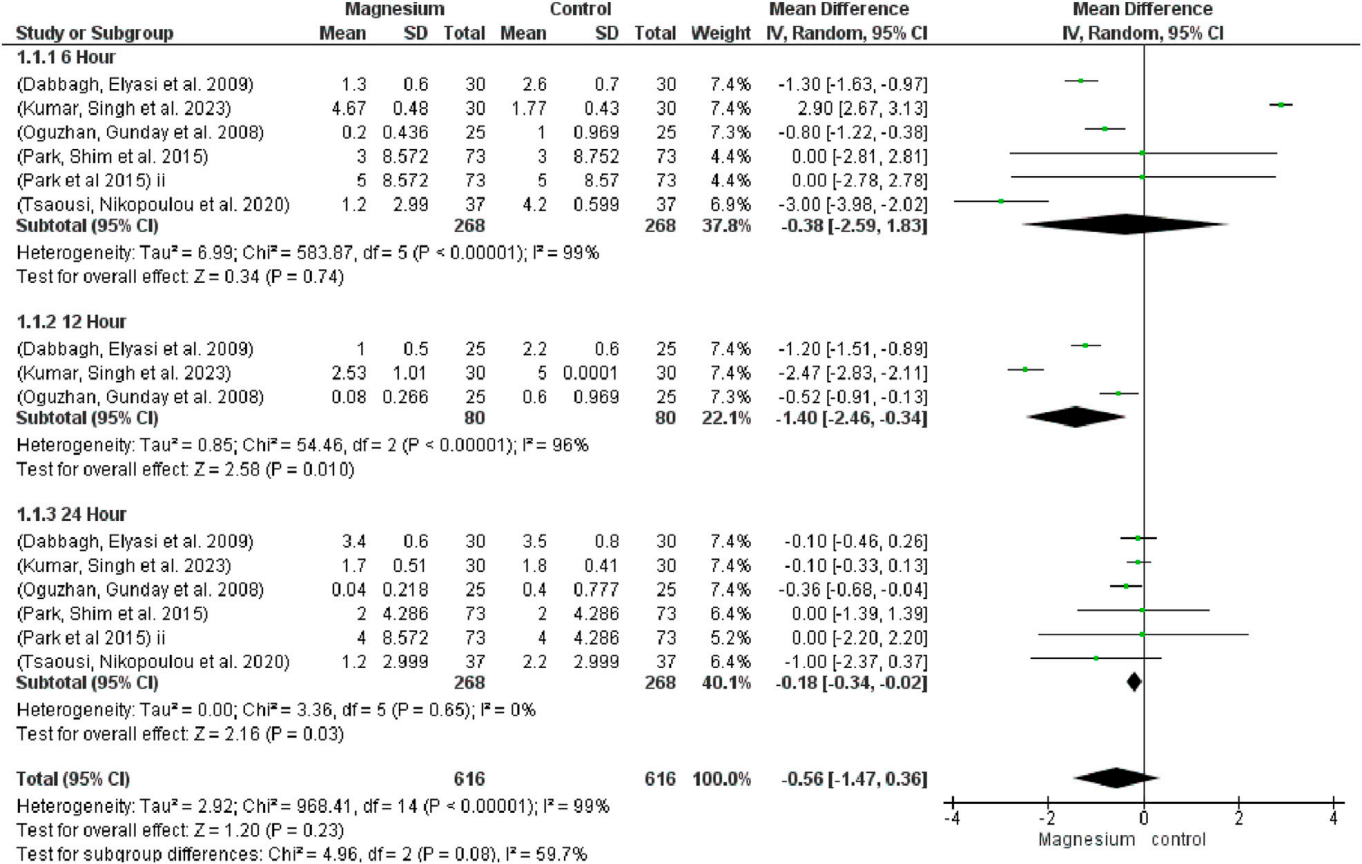
Forest plot displaying pain measured using Visual Analog Scale (VAS).

The global VAS (MD 0.56, 95% CI −1.47 to 0.36; participants = 1,232; studies = 15; I2 = 99%) showed no discernible differences (Figure 4). Comparably, at 6 h (MD −0.38, 95% CI −2.59 to 1.83; participants = 536; studies = 6; I2 = 99%) and 12 h (MD −1.40, 95% CI −2.64 to 0.34; participants = 160; studies = 3; I2 = 96%), no significant differences were discovered. After a full day, however, the magnesium group displayed noticeably less discomfort than the control group (MD−0.18, 95% CI−0.34, −0.02; participants, 536; studies, 6; I2 = 0%). When comparing MS with a placebo, MS showed a much higher global VAS reduction. There were no discernible changes between MS and dexmedetomidine. Likewise, no noteworthy distinctions were noted when contrasting MS with dexamethasone. The subgroup test is 1.20 and the overall effect test is 2.16.

#### 3.2.1 Drug consumption

**FIGURE 5:**
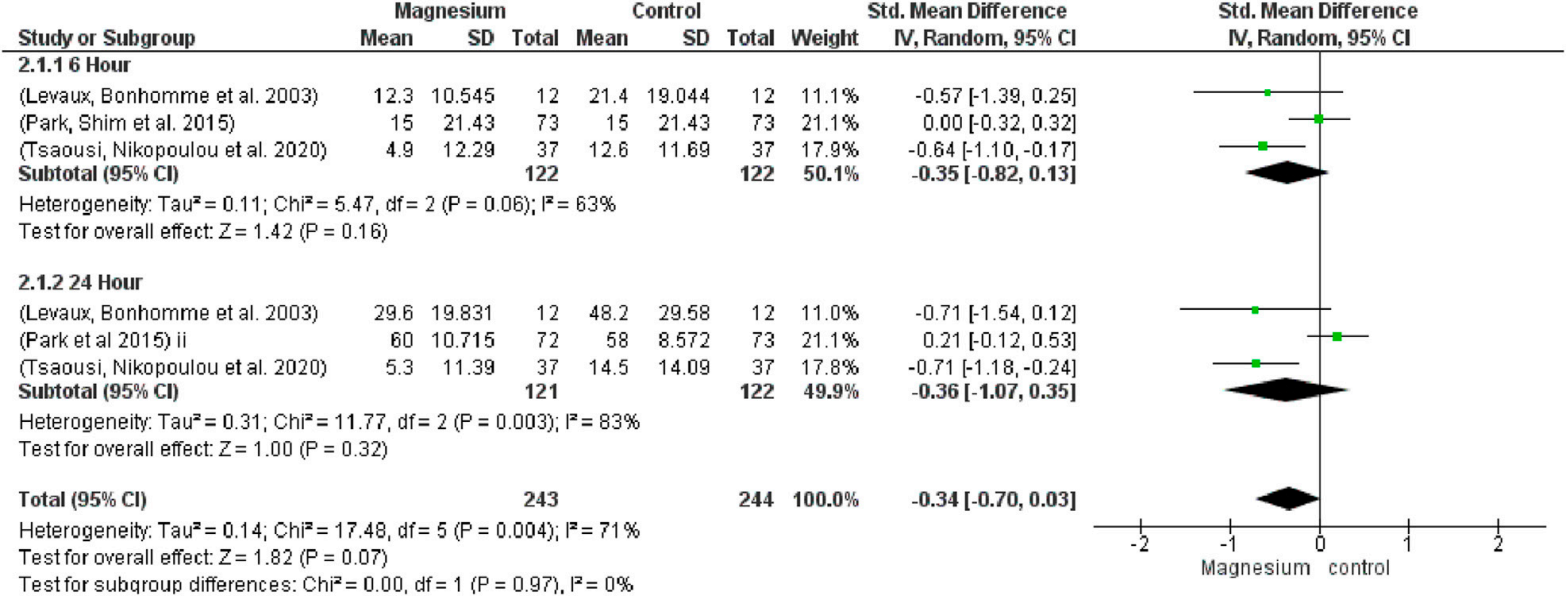
Forest plot of muscle relaxant consumption.

In relation to opioid consumption, no significant differences were observed at 6 h (SMD−0.35, 95%CI−0.82 to 0.13; participants = 244; studies = 3; I2 = 63%) or 24 h (SMD−0.36, 95% CI−1.07 to 0.35; participants = 243; studies = 3; I2 = 83%) (Figure 5). When magnesium was compared to a placebo, it was found to significantly reduce opioid consumption (SMD−0.66, 95%CI −0.95 to −0.38; participants = 196; studies = 6; I2 = 0%), both at 6 and 24 h (SMD−0.71, 95% CI −1.12 to −0.30; participants = 98; studies = 3; I2 = 0%). In terms of overall or at six or 24 h, there were no significant differences between MS and dexamethasone (SMD 0.10, 95% CI−0.13 to 0.33; participants = 292; studies = 6; I2 = 0%). Test results for the subgroup are 1.82 and the overall impact are 1.00.

**FIGURE 6:**
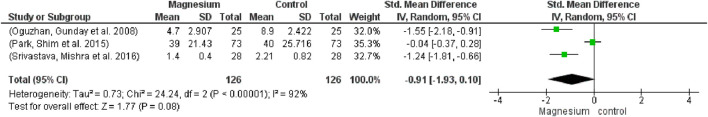
Forest plot of remifentanil consumption.

The MS group’s use of muscle relaxants was significantly lower among participants (252) and studies (3) (SMD −0.91, 95% CI−1.93 to −0.10; I2 = 92%) (Figure 6) MS had a significantly decreased consumption of muscle relaxants when compared to a placebo. The total impact test is 1.77 and the chi2 value is 0.73.

**FIGURE 7:**
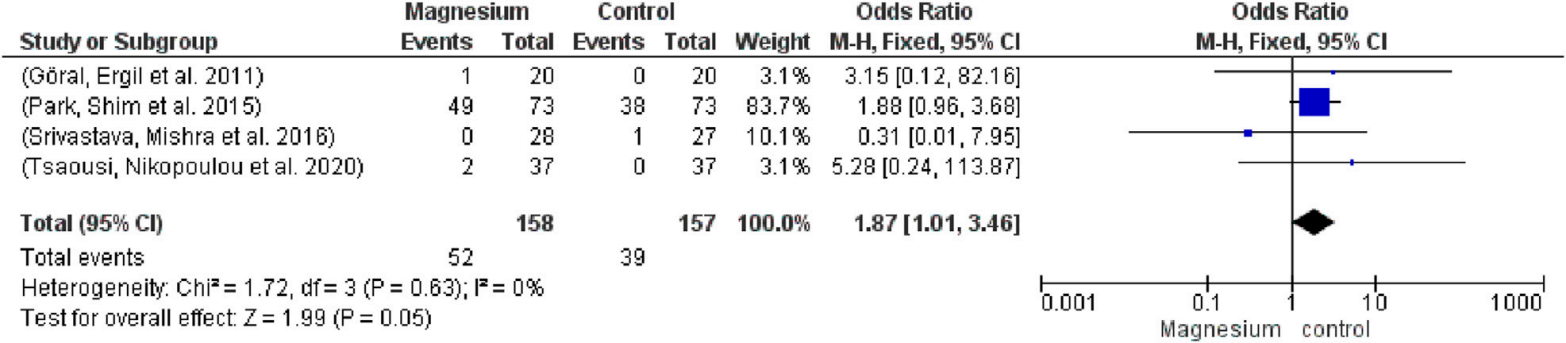
Forest plot of vasopressor consumption.

Vasoactive agent use did not differ significantly (OR 1.87, 95% CI 1.01 to 3.46; participants = 315; studies = 4; I2 = 0%) from (Figure 7). Not at all in contrast to a placebo. The chi2 value is 1.72 and the overall impact test is 1.99.

#### 3.2.2 Hemodynamics

**FIGURE 8:**
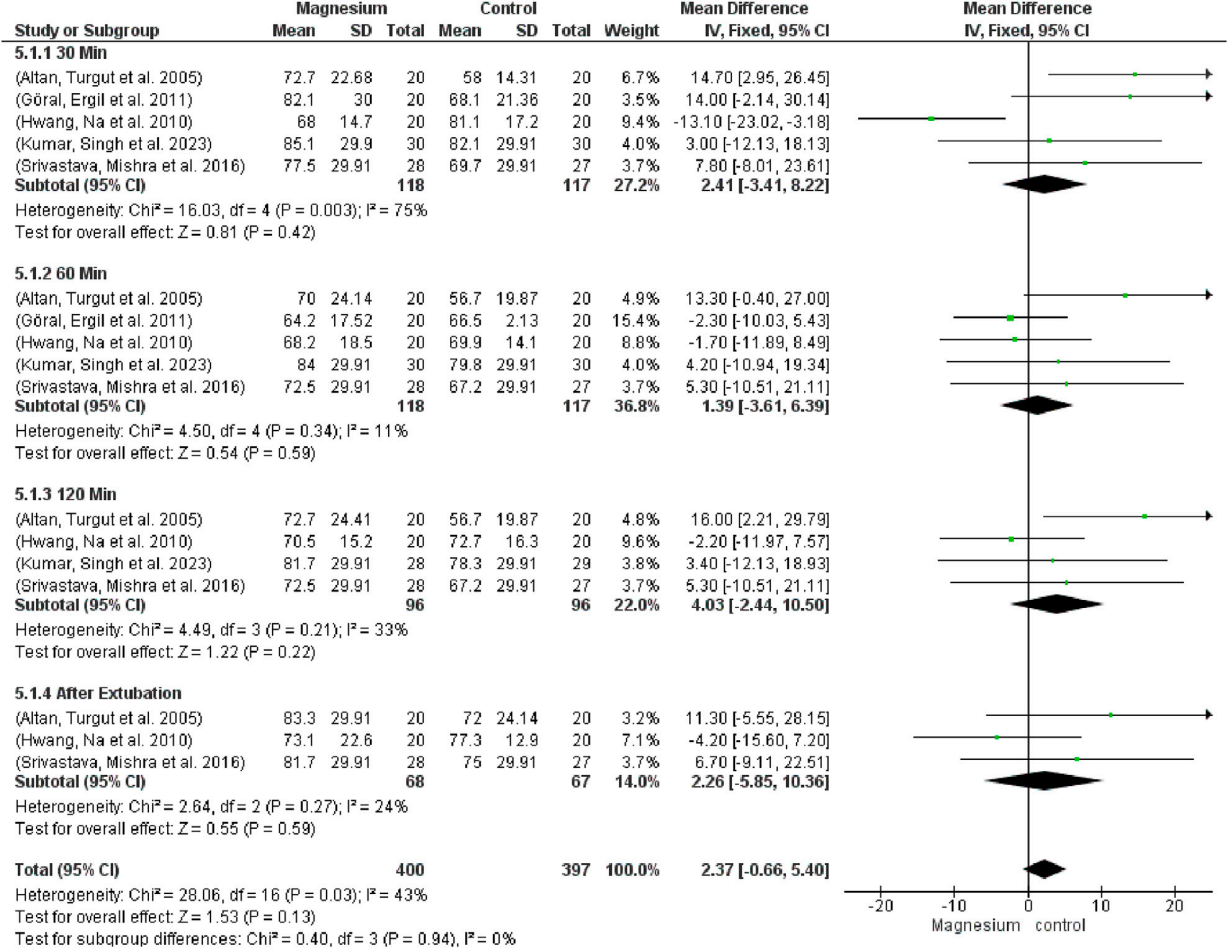
Forest plot results express heart rate at 30 min, 60 min, 120 min, and after extubating.

Regarding global heart rate (MD 2.37, 95% CI −0.66 to 5.10; participants = 797; studies = 17; I2 = 24%), there were no significant changes between the groups or in any of the follow-up time-based subgroups (Figure 8). The MS group displayed a considerably greater heart rate in comparison to the placebo. When MS was compared with clonidine, no discernible differences were found. Test results for subgroup difference are 0.40 and total effect are 0.55.

**FIGURE 9:**
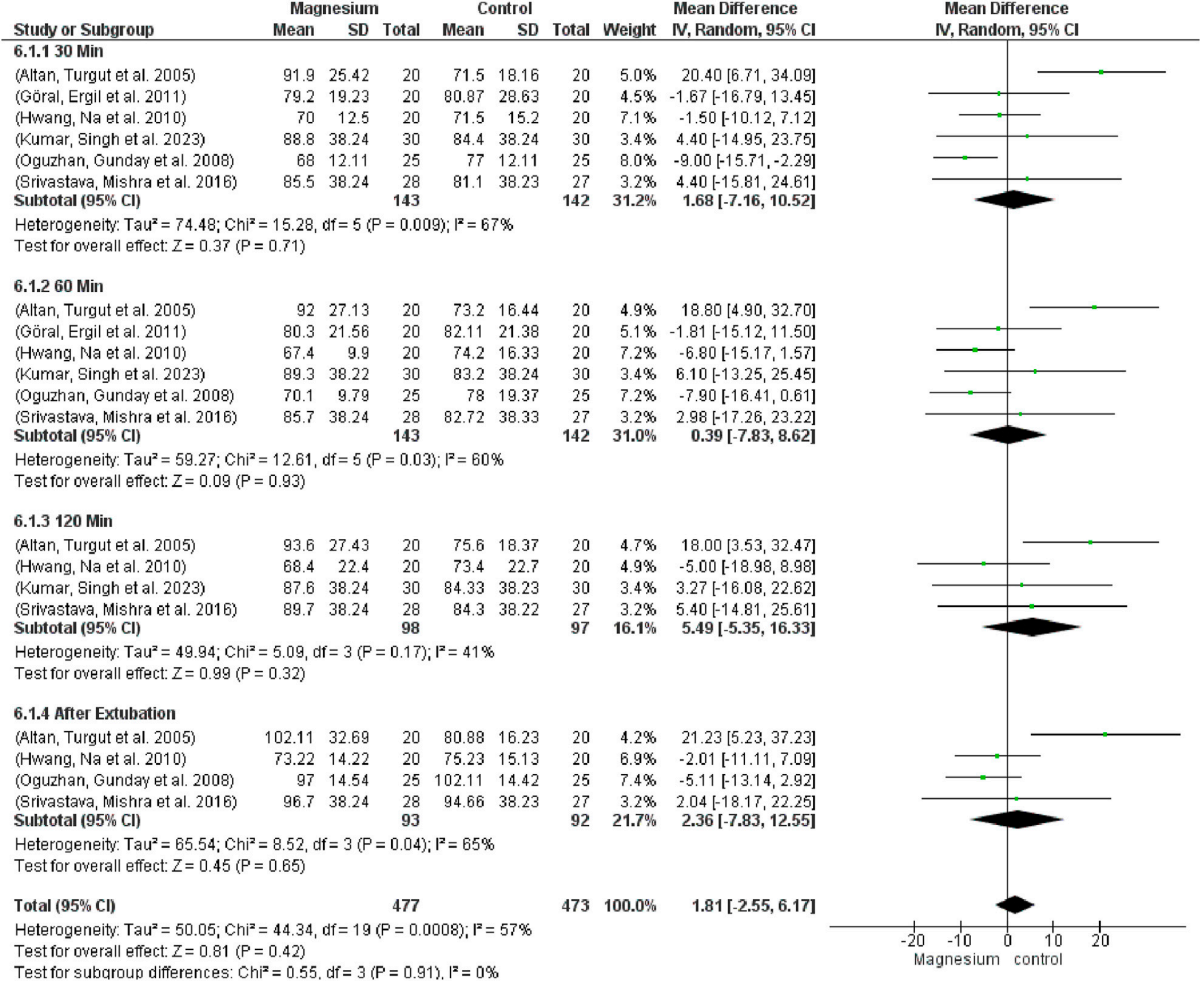
Forest plot express mean arterial pressure at 30 min, 60 min, 120 min, and after extubating.

Figure 9 indicates that there were no significant variations in mean arterial pressure (MAP) across the groups (MD 1.81, 95% CI−2.55 to 6.17; 950 participants, 20 studies, and 57% I2). Notably, there were no variations when compared to the placebo group. MS displayed a considerably decreased MAP in contrast to clonidine. Regarding dexmedetomidine, there were no notable variations. The subgroup test is 0.55 while the overall effect test is 0.45.

#### 3.2.3 Extubating time, response to verbal commands and orientation time

**FIGURE 10:**
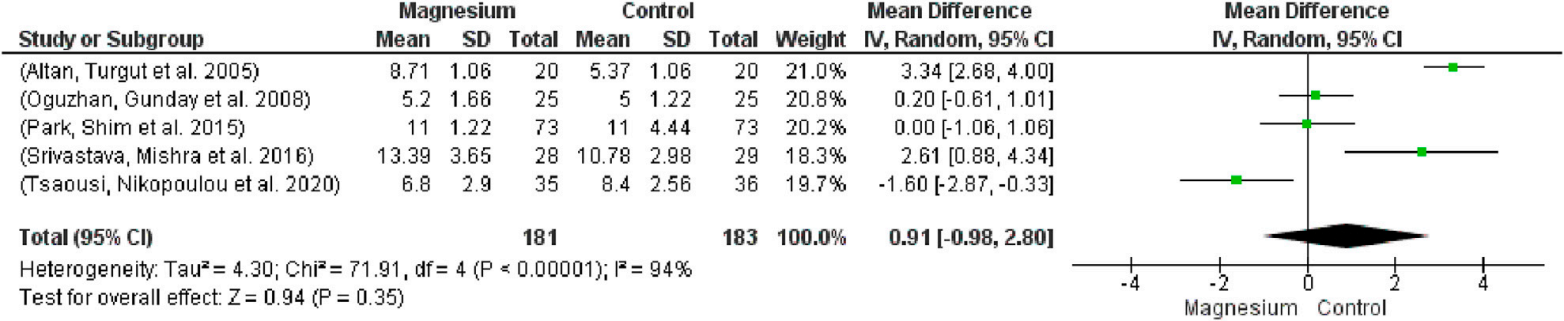
Forest plot of extubation time.

There was no statistically significant difference in extubating time between the groups (Figure 10; participants = 364, studies = 5, I2 = 94%; MD 0.91, 95% CI -0.98–2.80). With respect to the placebo group, there were no appreciable variations. The MS group had a considerably longer extubation time in comparison to dexmedetomidine. The overall effect test result is 0.94, and chi2 is 71.91.

**FIGURE 11:**
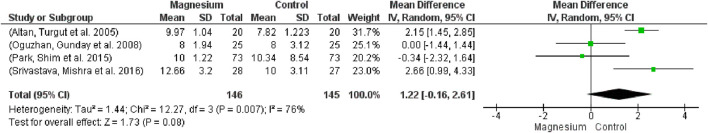
Forest plot of verbal commands.

Figure 11 shows that the MS group’s responsiveness to verbal orders was considerably higher (MD 1.22, 95% CI -0.166 to 2.61; participants = 291; studies = 4; I2 = 76%). With respect to the placebo group, there were no appreciable variations. In contrast to the clonidine group, the MS group displayed a noticeably slower response time to spoken instructions. Chi2 is 12.27 and the test for the total effect is 1.73.

**FIGURE 12:**
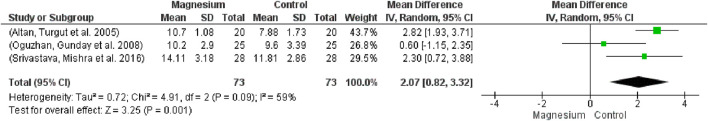
Forest plot of orientation time.

Additionally, the orientation time was substantially longer in the MS group compared to the placebo group (MD: 2.07, 95% CI 0.82 to 3.32; participants = 146; studies = 3; I2 = 59%) (Figure 12). The test effect was 3.25 overall, and the chi2 was 4.91.

#### 3.2.4 Publication bias

Visual inspection of funnel plots revealed significant publication bias across most variables, as evidenced by notable asymmetry in the distribution of studies. Detailed analysis of these asymmetries and their implications is presented in the supplementary materials.

The therapeutic benefits of magnesium in postoperative pain management likely stem from its multifaceted neuroprotective and anti-inflammatory mechanisms (Hassan et al., 2020). Through the promotion of neuroplasticity, magnesium provides protection against neuronal deterioration and postoperative cognitive dysfunction (Hassan et al., 2020). Its antioxidant properties are demonstrated through the neutralization of reactive oxygen species (ROS), specifically via hydrogen peroxide (H2O2) elimination and hydrogen (H2) liberation, contributing significantly to its anti-inflammatory effects in treating intervertebral disc degeneration (IVDD) (Hassan et al., 2020; Zhang et al., 2023).

The neuroprotective profile of magnesium extends to its modulation of S100B protein levels, a recognized biomarker of oxidative stress and amyloid precursor (Zhang et al., 2023). Clinical investigations have revealed that magnesium’s efficacy in managing postoperative sore throat is comparable to corticosteroids, potentially contributing to enhanced patient satisfaction. This finding is particularly noteworthy given that patient-reported outcome measures (PROMs) were primarily limited to Visual Analog Scale (VAS) assessments (Park et al., 2015). The comparison with corticosteroids merits special attention, considering their adverse metabolic effects, particularly on glucose homeostasis. The therapeutic benefit appears to be mediated through magnesium’s anti-inflammatory properties, resulting in reduced postoperative nausea, vomiting, and subsequent throat discomfort (Lu et al., 2013; Mori et al., 2010).

A significant limitation across studies was the absence of minimum clinically important difference (MCID) assessments, highlighting a crucial area for future investigation. The marked reduction in opioid consumption observed with magnesium administration, compared to placebo, can be mechanistically explained by its non-competitive N-methyl-D-aspartate (NMDA) receptor antagonism. This mechanism results in decreased glutamate release and inhibition of excessive calcium influx into neurons, thereby providing neuroprotection and maintaining cellular integrity (Newcomer and Krystal, 2001). Similar opioid-sparing effects have been documented with other NMDA receptor antagonists, including gabapentin and ketamine (Newcomer and Krystal, 2001).

The neuroprotective properties of magnesium sulfate have broader clinical applications, as evidenced by its recommended use in reducing cerebral palsy incidence among preterm infants, particularly those at risk of delivery before 32 weeks gestation (Jameson and Bernstein, 2019). Through dual mechanisms of neuronal process stabilization and vasodilation, intracerebral magnesium sulfate reduces the incidence of cerebral vasospasm and delayed cerebral ischemia. Complementary intravenous hydrogen therapy may enhance these effects through additional antioxidant benefits, potentially improving clinical outcomes and reducing cerebral oxidative stress (Takeuchi et al., 2021).

#### 3.2.5 Hemodynamic and cardiovascular effects

Our analysis revealed lower mean arterial pressure in the magnesium group compared to the corticosteroid group, attributable to magnesium’s vasodilatory properties. Magnesium exerts its hypotensive effect through multiple mechanisms: direct relaxation of blood vessels, calcium channel antagonism, and competition with sodium in vascular smooth muscle cells. Furthermore, magnesium enhances endothelial function and vascular wall integrity, promoting vasodilation and reducing inflammation (Houston, 2011). The observed increase in heart rate in our meta-analysis likely represents a compensatory response to this vasodilation, while the higher mean arterial pressure in the corticosteroid group can be attributed to adrenergic system activation.

The hypotensive properties of magnesium contribute to reduced perioperative bleeding (Rayssiguier et al., 2010). Additionally, magnesium modulates sympathetic nervous system activity through catecholamine blockade (Göral et al., 2011). While the magnesium group demonstrated prolonged activated partial thromboplastin time, other coagulation parameters remained comparable between groups (Wang et al., 2024).

#### 3.2.6 Neuromuscular effects and safety considerations

Magnesium’s ability to reduce muscle relaxant requirements is attributed to its inhibition of acetylcholine receptor responses in muscle cells. This synergistic effect potentially allows for dose reduction of muscle relaxants during surgical procedures. However, careful dose titration of both vecuronium and magnesium sulfate is essential to prevent adverse effects and excessive muscle relaxation (Aal-Hamad et al., 2023).

#### 3.2.7 Risk of hypermagnesemia

Careful monitoring of magnesium levels is crucial, as concentrations exceeding 4–5 mmol/L (9.7–12.2 mg/dL) are considered hazardous. Hypermagnesemia can manifest as weakness, nausea, dizziness, and confusion, with increased mortality risk in hospitalized patients. The prevalence ranges from 3.0% to 5.7%–9.3% in the general population, with higher rates in hospital settings. Patients with renal insufficiency require particularly vigilant monitoring and management to prevent serious complications (Srivastava et al., 2016).

#### 3.2.8 Recovery period considerations

The prolonged recovery period observed in the magnesium group, characterized by delayed orientation time and verbal response, likely results from the combination of its hypotensive and muscle-relaxant effects. The vasodilatory properties may lead to reduced cerebral perfusion and oxygenation, contributing to extended recovery times and temporary cognitive effects. The muscle-relaxant properties may further impair patient responses during the recovery phase, affecting both verbal communication and orientation capabilities.

#### 3.2.9 Comparative analysis with dexmedetomidine

Dexmedetomidine served as a key comparator in our meta-analysis, providing important insights into alternative therapeutic approaches. As an α2-adrenoceptor agonist, dexmedetomidine demonstrates significant efficacy in reducing surgical stress responses. Our analysis revealed that dexmedetomidine was more effective than magnesium in reducing both fentanyl and propofol consumption. Srivastav et al.'s findings demonstrated superior hemodynamic stability with dexmedetomidine compared to other interventions (Srivastava et al., 2016). This enhanced hemodynamic profile and greater efficacy in reducing opioid and propofol consumption can be attributed to dexmedetomidine’s high affinity and selectivity for α2-adrenergic receptors. However, these benefits are accompanied by an increased risk of bradycardia and hypotension compared to magnesium therapy (Srivastava et al., 2016).

#### 3.2.10 Pain management and recovery characteristics

While Kumar’s study reported superior pain control with dexmedetomidine compared to magnesium plus ropivacaine combination, our meta-analysis found no significant differences in pain outcomes between the interventions. Notably, we observed increased muscle relaxant consumption in the dexmedetomidine group. In comparison with placebo, dexmedetomidine demonstrated advantages in orientation time and verbal response parameters. The prolongation of local anesthetic effects observed with dexmedetomidine can be attributed to its vasoconstrictive properties (Yoshitomi et al., 2008).

#### 3.2.11 Safety profile and administration considerations

Recent meta-analyses have highlighted specific safety concerns with dexmedetomidine administration during spinal surgery. The risk of bradycardia and intraoperative hypotension is particularly pronounced when administered as a loading dose in combination with total intravenous anesthesia (Wang et al., 2024). These risks were notably elevated in patients receiving inhalation anesthesia. While the inhalation anesthesia subgroup demonstrated reduced blood loss, this effect was not observed consistently across all administration methods (Wang et al., 2024).

#### 3.2.12 Multimodal applications and synergistic effects

Magnesium demonstrates significant potential for synergistic effects when combined with other analgesic modalities. The modest effects observed in comparisons between magnesium sulfate and corticosteroids may be attributed to limited sample sizes and short follow-up periods (typically 24 h). Evidence suggests enhanced patient outcomes when magnesium is combined with various analgesic interventions, including opioids, local anesthetics, and regional anesthesia techniques (Fairley et al., 2017). This multimodal approach may provide more comprehensive pain management strategies, though longer-term monitoring beyond the immediate postoperative phase is needed to fully evaluate these benefits.

#### 3.2.13 Administration protocols and dosing strategies

Considerable variation exists in magnesium administration protocols (Tsaousi et al., 2020). Our meta-analysis evaluated various approaches, including:

• Loading doses: 30–50 mg/kg in saline, administered over 10–30 min pre-anesthesia.
• Maintenance infusions: 10–20 mg/kg/h throughout surgery.
• Alternative approaches: One study evaluated ropivacaine infiltration with 500 mg magnesium supplementation.

While visual analysis of forest plots did not reveal clear associations between administration protocols and outcomes, this observation requires cautious interpretation given the lack of formal statistical analysis.

#### 3.2.14 Broader clinical applications

Magnesium’s therapeutic benefits extend beyond spinal surgery. Evidence demonstrates its efficacy in.

• Reducing postoperative atrial fibrillation in cardiac surgery (Fairley et al., 2017).
• Decreasing ventricular arrhythmias without additional adverse effects.
• Improving postoperative pain control in arthroscopic procedures when administered intra-articularly.
• Providing cartilage and chondrocyte protection, as supported by experimental studies (Zeng et al., 2016).

Meta-analyses have documented significant reductions in:

• 24-h morphine consumption.
• Time to first analgesic requirement.
• Postoperative shivering without increasing adverse effects such as bradycardia, nausea, or vomiting (Ng et al., 2020).

#### 3.2.15 Surgical outcome analysis

Our study expanded upon previous meta-analyses by incorporating additional outcome measures, including remifentanil and muscle relaxant consumption. Key findings include.

• Peak pain reduction occurred at varying timepoints (6–12 h) across studies (Tsaousi et al., 2020; Kumar et al., 2023).
• Tsaousi et al. uniquely reported decreased extubation time with corresponding reductions in opioid and remifentanil use (Tsaousi et al., 2020).
• Variable outcomes in specific surgical subgroups:
o Microscopic surgery (Göral et al. (2011)): Limited comparable outcomes.
o Lumbar disc replacement: Significant improvements in pain control and reduced muscle relaxant requirements (Kumar et al., 2023).
o Broader surgical categories (“lumbar arthrodesis,” “spine surgery”): Limited comparative analysis due to heterogeneity (Morrison et al., 2013).

Unlike previous meta-analyses focusing on total anesthesia duration, our study specifically examined orientation and verbal response times, though findings aligned with established observations regarding prolonged anesthetic effects.

## 4 Limitations

There are several restrictions on this study. The restricted number of included studies hindered the ability to do sensitivity and consistent subgroup analysis, as well as to assess the impact of varying doses and surgical indications. To give more detailed advice, future research should describe findings according to the precise kind of surgery that was done. Additionally, rather than using formal procedures, publication bias was evaluated visually, and in several instances, there were just a few publications available for the control groups. Even though the established Cochrane guidelines were adhered to, challenges pertaining to missing data were also observed. Furthermore, several intriguing factors, including improved hemostatic parameters, decreased intraoperative bleeding, and patient satisfaction, were only reported in one trial and could not be compared via meta-analysis. Data on these characteristics should be used in future research. These restrictions should be considered when interpreting the findings, and they highlight the need for more study to address these restrictions in order to gain a more thorough understanding of the application of magnesium in the treatment of postoperative pain.

## 5 Conclusion

In conclusion, our meta-analysis showed that magnesium sulphate delivery following spinal surgery significantly reduced pain at 24 h and minimized the use of opioids and muscle relaxants when compared to placebo and other analgesics. Magnesium sulphate also extended orientation and reactions to spoken instructions. The groups’ heart rates and blood pressure did not differ significantly from one another. Without causing greater side effects, this multimodal strategy using magnesium sulphate seemed to reduce postoperative pain more well. These results imply that magnesium sulphate may improve recovery regimens optimised after spine surgery and solve current problems associated with opioid use. It is yet unknown how clinical improvements will translate to patient outcomes and what effect they will have on hospital stays, patient satisfaction, and care quality.

## Data Availability

The original contributions presented in the study are included in the article/Supplementary Material, further inquiries can be directed to the corresponding author.
