# Supplementary material for: Effectiveness and impact of intravenous magnesium sulfate in spinal surgery systematic review and meta-analysis
Source: Front Pharmacol. 2025 Jun 18;16:1624119. doi: 10.3389/fphar.2025.1624119 (PMC12213652; doi:10.3389/fphar.2025.1624119)
Supplement: Supplementary file 1 [file Supplementaryfile1.docx]

**Search Strategy of different databases:**

Search: magnesium sulphate AND spine ("magnesium"[MeSH Terms] OR "magnesium"[All Fields] OR "magnesiums"[All Fields] OR "magnesiums sulphate"[All Fields]) AND ("spine"[MeSH Terms] OR "spine surgery"[All Fields] OR "spines"[All Fields] OR "spine s"[All Fields]) Translations magnesium: "magnesium"[MeSH Terms] OR "magnesium"[All Fields] OR "magnesium's"[All Fields] OR "magnesiums"[All Fields] spine: "spine"[MeSH Terms] OR "spine"[All Fields] OR "spines"[All Fields] OR "spine's"[All Fields]

**Publication bias assessment:**

**Pain:**

**
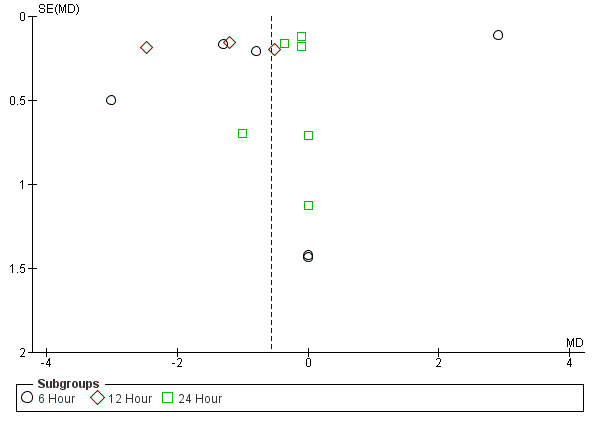
**

**Opioids consumption:**

**
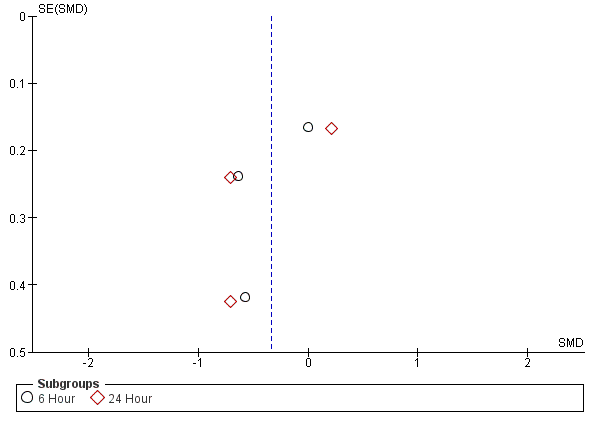
**

**Hemodynamics**

**
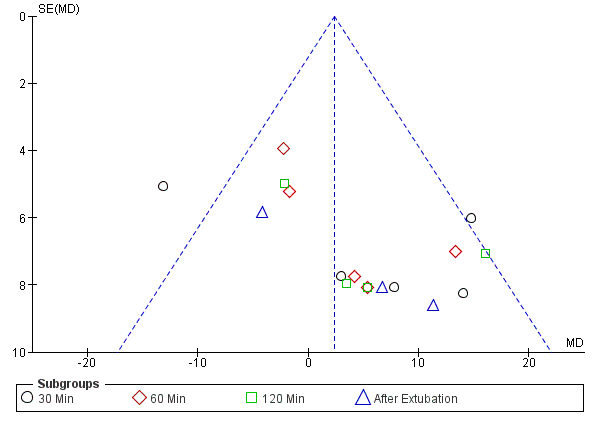
**

**Mean Atrial pressure:**

**
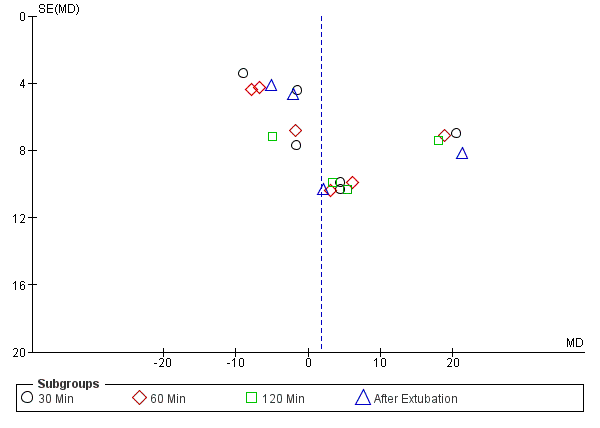
**
